# Impact of Dietary Betaine and Metabolizable Energy Levels on Profiles of Proteins and Lipids, Bioenergetics, Peroxidation and Quality of Meat in Japanese Quail

**DOI:** 10.3390/ani11010117

**Published:** 2021-01-08

**Authors:** Sabry M. El-Bahr, Saad Shousha, Wasseem Khattab, Ahmed Shehab, Osama El-Garhy, Hoda El-Garhy, Shereen Mohamed, Omar Ahmed-Farid, Ahmed Hamad, Islam Sabike

**Affiliations:** 1 Department of Biomedical Sciences, College of Veterinary Medicine, King Faisal University, P.O. Box 400, Al-Ahsa 31982, Saudi Arabia; smmohamed@kfu.edu.sa; 2 Department of Biochemistry, Faculty of Veterinary Medicine, Alexandria University, Alexandria 21526, Egypt; 3 Department of Physiology, Faculty of Veterinary Medicine, Benha University, Benha 13736, Egypt; 4 Department of Nutrition and Clinical Nutrition, Faculty of Veterinary Medicine, Benha University, Moshtohor, Qalioubia, Benha 13736, Egypt; wasim.hassan@fvtm.bu.edu.eg (W.K.); ahmed.shehab@fvtm.bu.edu.eg (A.S.); 5 Department of Animal Production, Faculty of Agriculture, Benha University, Moshtohor, Qalioubia, Benha 13736, Egypt; osama.elsayed1977@yahoo.com; 6 Department of Genetics, Faculty of Agriculture, Benha University, Moshtohor, Qalioubia, Benha 13736, Egypt; hoda.algarhy@fagr.bu.edu.eg (H.E.-G.); shereen.mustafa@fagr.bu.edu.eg (S.M.); 7 Department of Physiology, National Organization for Drug Control and Research, Giza 12622, Egypt; ebntaimya@yahoo.com; 8 Department of food Hygiene, Faculty of veterinary medicine Benha University, Benha 13736, Egypt; dr.ahmedalhosieny@gmail.com (A.H.); islam.sabek@fvtm.bu.edu.eg (I.S.)

**Keywords:** betaine, Japanese quail, energy, lipids, meat

## Abstract

**Simple Summary:**

Three hundred and sixty quails were divided into six groups to evaluate the effect of dietary betaine on profiles of proteins and lipids, bioenergetics, peroxidation and quality of meat of Japanese quail fed diets containing three levels of metabolizable energy (ME) viz. optimum (2900 kcal ME/kg), restricted (2800 kcal ME/kg) and low (2700 kcal ME/kg). Dietary betaine improved meat quality parameters viz. cooking loss, thawing loss and water-holding capacity of Japanese quail fed diets containing either restricted or low ME by enrichments of the meat with omega-3 fatty acids and reduction of lipids levels.

**Abstract:**

Three different diets were formulated with three levels of metabolizable energy (ME) (optimum; 2900, restricted; 2800 and low; 2700 kcal ME/kg diet) without or with (0 and 0.15%) betaine supplementation in 2 × 3 factorial design to evaluate the effect of six experimental diets on performance, proteins and lipids profiles, bioenergetics, peroxidation and meat quality of Japanese quail. Therefore, 360 quails allocated into six groups in a 23-day experiment. Dietary betaine and ME levels did not affect the performance, meat energy indices (ATP and AMP) and malondialdehyde (MDA) levels of Japanese quail meat. Dietary betaine and/or ME levels induced significant changes in serum triacylglycerol (TAG), total cholesterols (TC), low-density lipoprotein cholesterol (LDL-c), very low-density lipoprotein cholesterol (VLDL-c), meat total lipids and cholesterol of Japanese quail. Optimum and restricted ME levels reduced total volatile basic nitrogen (TVBN) whereas dietary betaine increased ecosapentaenoic (EPA), docosahexaenoic acids (DHA) and glutamine concentrations in breast meat of Japanese quail. Dietary betaine and low energy diet improved cooking loss, thawing loss (ThL) and water holding capacity (WHC) in breast meat of Japanese quail. Conclusively, dietary betaine improved meat quality of Japanese quail fed diets containing either restricted or low ME by enrichments the meat with omega-3 fatty acids and reduction of lipids levels.

## 1. Introduction

Japanese quails (*Coturnix coturnix japonica*) have early sexual maturity, rapid growth rate and small body size. quails’ meat is highly recommended for human due to its low fat and cholesterol contents [1]. Betaine (trimethylglycine) provides a better perspective in animal nutrition. Dietary betaine has received a great attention due to its antioxidant [2,3], osmo-protectant [4,5] and methionine-sparing properties [6]. Betaine was widely used in animal nutrition as methyl donor during methionine metabolism [7]. Its labile methyl groups involved in the transmethylation reactions for various metabolic processes [8]. Therefore, it had a great impact on both protein and energy metabolism. For instance, dietary betaine reduced fat and increased lean meat content of broiler meat [6,9]. In this regards, many research studies have been reported an increase in breast meat yield a long with a significant reduction in abdominal fat content of broilers [10], ducks [11] and turkeys [12]. The methionine-sparing activity of betaine is the main reason for extra protein biosynthesis [13] and hence, is highly effective not only to increase breast meat yield but also to reduce dietary need for methionine in poultry [6]. Recently, researchers’ attention has been directed to innovate the appropriate methods for improvement of meat quality [14] to meet the customer needs and preference [9]. Dietary betaine improved the meat quality of broiler chickens by reducing lipid peroxidation [2,15]. Dietary betaine reduced the detrimental effects of long-term heat stress on growth performance, digestive function and carcass traits in indigenous yellow-feathered broilers [16]. Energy supplements in poultry diets represent a major cost in the poultry industry. Besides, these supplements have significantly increased the unfavorable abdominal and muscular fat content in modern chicken breeds. Thus, innovative feeding approaches are required to reduce the overall cost of poultry production possibly, by decreasing dietary energy concentrations while, maintaining optimum or even higher performance [17]. Scarce literatures have been dealt with the energy matrix of betaine in poultry diets [18].These articles outlined that dietary betaine provided an energy matrix spare of 200 KJ/kg diet. The lipid modulating activity of betaine could effectively perform such task especially if we would hypothesize an experimental design with low-energy diets. We hypothesized that dietary betaine could affect both energy and protein metabolism as well as alter meat quality in low-energy diets. Therefore, this study aimed to evaluate effect of dietary betaine on profiles of proteins and lipids, bioenergetics, peroxidation and quality of meat of Japanese quail fed diets containing different levels of ME viz. optimum (2900 kcal ME/kg), restricted (2800 kcal ME/kg) and low (2700 kcal ME/kg).

## 2. Materials and Methods

### 2.1. Animal Management, Diets and Experimental Design

All bird management procedures, handling and sampling as well as sacrificing for the experiment were approved by the Institutional Animals Care and Use Committee Research Ethics of Benha University, Faculty of Veterinary Medicine (BUFVTM, 10/01/2018), Egypt. Three hundred and sixty Japanese quail chicks of both sexes brought from commercial hatchery were used in this study at Poultry Farm, Faculty of Agriculture, Benha University, Egypt. Quails at 12-day old were weighed (45.50 ± 0.072) and then divided into six equal groups (60 quails each) of three replicates (20 quails each). Three different diets were formulated with 3 levels of metabolizable energy (optimum; 2900, restricted; 2800 and low; 2700 kcal ME/kg diet) without or with (0 and 0.15%) synthetic betaine (Skystone Feed Co. Ltd., Jiangsu, China) in 2 × 3 factorial design (Table 1). Optimum diets either without or with betaine were formulated to achieve the optimum nutrient requirements of growing Japanese quails according the guidelines of National Research Council [19]. Quails were placed in galvanized cages (89 × 40 × 30 cm, length × width × height), supplied with feeders and nipple drinkers and were allowed for free choice access to mash feed (ad libitum) and water in an entirely randomized design for 23 days period till the end of the experiment (35th day of age).

### 2.2. Growth Performance

During the experiment, the following records were routinely registered. Weekly amount of offered and rejected feed for each cage (i.e., replicate) to calculate the feed intake (FI) per bird [20]. Weekly quail’s weight for each cage to calculate the average weight and then the weight gain (WG) per bird. Feed conversion ratio (FCR) per bird was calculated dividing FI by WG [21]. Birds have been fasted 8 h before weighing.

### 2.3. Blood and Meat Sampling 

At the end of experiment, five birds from each replicate (15 quails/group) were selected randomly to be slaughtered for sampling. The birds were bled out for 2 min and blood samples (approximately 4 mL) were collected then centrifuged at 3000 rpm for 15 min to separate serum. 

Pectoralis minor and pectoralis major muscles were excised from quail carcasses and were directly chilled at 4 °C for 24 h for subsequent analysis of meat quality parameters, including pH, WHC and Cooking loss and chemical parameters, including moisture and ash percentages, while another part was weighed and stored at −18 °C for one week for ThL estimation.

Birds have been fasted 8 h before samples collection.

### 2.4. Analyses of Serum Biochemical Parameters

Serum HDL-c, LDL-c and VLDL-c as well as TAG were determined calorimetrically by using commercial kits (Stanbio Laboratory Company; Boerne, TX 78202, USA). 

### 2.5. Estimation of Meat Quality Parameters

Total volatile basic nitrogen (TVBN) was determined in breast meat samples by distillation (Micro-Kjeldahl technique; Vapodest 30 S distillation unit, Gerhardet, Germany) [22]. Breast meat samples were weighed, prepared and homogenized following the protocol outlined by Ahmed-Farid, et al. [23] for quantification of malondialdehyde (MDA), Adenosine triphosphate (ATP), Adenosine diphosphate (ADP) and Adenosine monophosphate (AMP), using high performance liquid chromatography (HPLC; Agilent HP 1200 series apparatus, Santa Clara, CA, USA) following the method outlined by Abd-Elrazek and Ahmed-Farid [24].

Tissue homogenate of breast meat samples was prepared and centrifuged (4000 rpm for 15 min). The obtained supernatant was used for the determination of total lipids and total cholesterol by colorimetric method following the manufacturer’s instructions using commercial kits (Stanbio Laboratory Company; Boerne, TX 78202, USA). Total lipids were extracted from breast meat samples using chloroform:methanol (2:1; *v*/*v*) solution, vortexed for 2 min and then centrifuged for 10 min at 1792× *g* [25]. Fatty acid methyl esters (FAME) were prepared from the supernatant using methanol:sulphuric acid mixture (95:5; *v*/*v*) and hexane following the esterification process [26]. Hexane extract of FAME was injected into gas chromatography (GC; Agilent Technologies 7890A, Santa Clara, CA, USA) equipped with SP2330 column (30 mm × 0.32 mm × 0.2 μm film thickness; Supelco Analytical, Bellefonte, PA, USA) and flame ionization detector using a temperature gradient program with hydrogen as carrier gas and split model [27]. FAME peaks were identified by comparison with retention time of fatty acids standard (Cat. No. 24073, Sigma-Aldrich, St. Louis, MO, USA) using Hewlett-Packard ChemStation software (Agilent Technologies Inc., Wilmington, DE, USA). Breast meat samples were prepared, homogenized, centrifuged and filtrated [26]. Filtrate was derivatized [28]. Derivatized meat samples as well as amino acid standards (Sigma-Aldrich, St. Louis, MO, USA) were injected into HPLC (Agilent HP 1200 series apparatus, Santa Clara, CA, USA) equipped with Nova-PakTM C18 column (4 μm, 3.9 × 4.6 mm) for separation and quantification of free amino acids as described earlier [29] with some modifications [26]. Ultimate pH (pH_U_), water-holding capacity (WHC), cooking loss and thawing loss (ThL) were measured in breast meat fillet. At 24 h postmortem, the final pH (pH_U_) was measured with a portable pH meter (Jenway 3510 pH-meter, Cole-Parmer, Staffordshire, United Kingdom) in the pectoralis major muscle at about 1-cm depth [30]. WHC was measured by the low-speed centrifugation methods [31], with a little modification. Briefly, a 5 g of intact meat sample was centrifuged in 15 mL falcon tube containing glass beads at 10.000× *g* and 5 °C for 20 min, then the meat was removed directly by forceps and dried with filter paper and reweighed. WHC was expressed as a percentage of difference in meat weight before and after centrifugation. Cooking loss (CL) was conducted on the previously weighed breast meat samples. These samples were placed individually in thermo-tolerant plastic bags and incubated in a preheated water bath for 6 min, a time enables muscle core temperature to reach 70 °C. Samples were cooled in ice water for 15 min. The cooled samples were taken from the bag, blotted dry, and reweighed. Cooking loss was calculated as a percent of initial uncooked weight [32]. To estimate ThL, the breast meat samples were initially weighed before holding frozen at –18 °C and weighed after thawing; at 5 °C for 24 h. Total loss was calculated as the weight difference between frozen and thawed ThL expressed in a percentage. Proximate dry matter (DM) was measured by oven drying while, moisture percentage was calculated by mass difference (100–DM%). Ash content was measured by combustion in muffle furnace following the standard methods of AOAC [33].

### 2.6. Statistical Analysis 

The recorded data were analyzed using two-way analysis of variance (ANOVA) as 2 × 3 factorial arrangements (2 levels of betaine × 3 levels of ME). The cage means (individual replicate) were considered as experimental units. The model was designed to analyze both means (i.e., Betaine and ME) as well as interaction (Betaine × ME) effects. The general linear model (GLM), Univariate of IBM SPSS statistics 19 was performed for all analysis. Duncan post hoc test was used for multiple comparisons among the three energy levels with a confidence level at (*p* ≤ 0.05). The *p* values having a significant interaction (Betaine × ME) were re-analyzed using one-way ANOVA for multiple comparisons with a confidence level at (*p* ≤ 0.05). 

## 3. Results

### 3.1. Feed Ingredient and Nutrient Compositions of Experimental Diets 

Percentage composition of feed ingredient and nutrient calculated analysis of the six experimental diets formulated for Japanese quail are presented in Table 1. As anticipated, the calculated values for the three levels of ME (2900, 2800 and 2700 kcal/kg diet), (optimum, restricted and low) for betaine-non-supplemented and betaine-supplemented diets, respectively were obtained properly (Table 1) to test our hypothesis. The same level of crude protein (CP) was achieved throughout the six experimental diets as designed (Table 1). 

### 3.2. Performance of Japanese Quail

The effect of dietary betaine supplementation and energy levels on Japanese quail performance is presented in Table 2. WG, FI and FCR remained unchanged significantly in Japanese quails fed diet of optimum, restricted, and low ME without (0%) or with betaine (0.15%) (Table 2).

### 3.3. Determination of Serum Lipids Profile

Effect of dietary betaine supplementation and different energy levels on serum lipids is shown in Table 3. Dietary betaine supplementation had significantly reduced serum TAG (*p* = 0.0013) and total cholesterol (TC) (*p* = 0.005) levels particularly in birds fed diets with restricted and low ME levels compared with birds fed betaine-non-supplemented diets with optimum and restricted ME levels. Similarly, dietary ME levels at reduced levels have significant reduction on the values of serum TAG and TC (*p* < 0.0001) levels of birds fed diet without or with betaine supplementation compared with birds fed betaine-non-supplemented diet with optimum ME level. Furthermore, significant reduction in serum TAG and TC levels (*p* < 0.0001) was noticed particularly in birds fed betaine-supplemented diet with restricted ME level compared to that of birds fed betaine-non-supplemented diet at the same level of energy (2800 kcal ME/kg diet). Decreasing dietary ME level reduced the values of serum HDL-c linearly (*p* < 0.0001) irrespective to betaine supplementation. On the contrary, betaine supplementation did not have significant effect on serum HDL-c (*p* = 0.106) of betaine-supplemented birds relative to their corresponding controls. Similarly, betaine supplementation did not have significant effect on serum LDL-c and VLDL-c levels (*p* = 0.064) of betaine-supplemented birds relative to their corresponding controls. Serum LDL-c and VLDL-c concentrations were significantly (*p* < 0.01) increased in birds fed restricted energy diet without betaine and were decreased significantly in birds fed low energy diet without betaine, restricted energy diet with betaine and low energy diet with betaine compared to that of birds fed betaine-non-supplemented diet with an optimum ME level. The significant increase in serum LDL-c and VLDL-c (*p* < 0.0003) was noticed especially in birds fed betaine-supplemented diets compared to that of birds fed betaine-non-supplemented diets at the optimum level of ME (2900 kcal ME/kg diet).

### 3.4. Bioenergetics, Lipid Peroxidation and Protein Breakdown of Breast Meat

The effect of dietary betaine supplementation and energy levels on cell energy, lipid peroxidation and protein breakdown of breast meat of Japanese quail are presented in Table 4. Irrespective to dietary ME levels, dietary betaine did not affect the levels of ATP, AMP, MDA and TVBN levels significantly (*p* > 0.05) in breast meat of Japanese quail. ADP concentrations were increased significantly (*p* = 0.042) in breast meat of birds fed betaine-supplemented diets compared to that of birds fed betaine-non-supplemented diets. ATP, ADP, AMP and MDA in the breast meat of Japanese quail were not affected significantly (*p* > 0.05) by the changes in ME levels. TVBN level of breast meat was significantly reduced (*p* = 0.009) only in birds fed betaine-supplemented diet with an optimum and restricted ME levels compared with that of birds fed betaine-non-supplemented diet with an optimum and low ME level. Breast meat MDA was significantly reduced (*p =* 0.002) in birds fed betaine-supplemented diet containing an optimum level of ME compared to that of birds fed betaine-supplemented diet containing low level of ME and that of birds fed betaine-non-supplemented diet containing the restricted ME level. 

### 3.5. Breast Meat Fat, Cholesterol and Fatty Acid Compositions 

Effect of dietary betaine supplementation and energy levels on breast meat fat, cholesterols and fatty acid compositions of Japanese quail is presented in Table 5. Dietary betaine had significantly reduced breast meat total lipids (*p* = 0.009) of birds fed diets containing low ME level compared with that of birds fed either betaine-supplemented or betaine non-supplemented diets containing optimum and restricted ME levels (*p* = 0.006). Breast meat cholesterol was significantly reduced (*p* = 0.017) in birds fed betaine-supplemented diets with an optimum, restricted and low ME levels compared with that of birds fed betaine-non-supplemented diet with optimum ME level. Moreover, noticeable reduction in breast meat cholesterol in a decreasing manner (*p* = 0.012) was observed when dietary ME level was reduced from optimum to restricted and then to low within betaine-non-supplemented diets. Dietary betaine and dietary ME levels had no significant effects on palmitoleic, linoleic, linolenic and arachidonic acids concentrations in breast meat of Japanese quail. Ecosapentaenoic (EPA) and docosahexaenoic acids (DHA) concentrations were increased significantly (*p* < 0.0001 and *p* = 0.0002) in meat of birds fed betaine supplemented diets compared to that of birds fed betaine-non-supplemented diets.

### 3.6. Breast Meat Amino Acid Compositions 

Effect of dietary betaine supplementation and ME levels on breast meat amino acid composition of Japanese quail is shown in Table 6. For essential amino acids, lysine was significantly lower (*p =* 0.017) in breast meat of birds fed betaine-supplemented diets compared to that of birds fed betaine-non-supplemented diets. On the other hand, for non-essential amino acids, glutamine levels were considerably higher (*p* < 0.0001) in meat of birds fed betaine-supplemented diets than that of birds fed betaine-non-supplemented diets.

### 3.7. Physicochemical Characters of Breast Meat

Effect of dietary betaine supplementation and energy levels on physicochemical characters of breast meat of Japanese quail is shown in Table 7. Dietary betaine did not significantly alter all studied meat physicochemical parameters (pH_U_, WHC, CL, ThL, moisture percentage and ash percentage). Dietary ME levels did not affect pH_U_, CL, ThL and ash percentage while, had a pronounced significant effect on WHC (*p* < 0.0001) and moisture percentage (*p* < 0.004) of breast meat of Japanese quail. WHC and CL were significantly increased (*p* < 0.0001; *p* < 0.021) in breast meat of birds fed betaine-supplemented diet containing low ME level compared with that of birds fed betaine supplemented diets containing restricted or optimum ME levels and that of birds fed betaine-non-supplemented diets containing all studied ME levels. ThL was significantly higher in breast meat of birds fed betaine-non-supplemented diet containing low ME level compared with that of birds fed betaine-non-supplemented diets containing optimum and restricted ME levels. Dietary betaine significantly reduced ThL (*p* < 0.005) in breast meat of birds fed diet containing low ME level compared with that of birds fed betaine-non-supplemented diet containing the same ME level. Moisture percentage was significantly reduced (*p* < 0.004) in breast meat of birds fed low energy diets irrespective to betaine supplementation compared with that of birds fed either betaine-supplemented or betaine-non-supplemented diets containing optimum and restricted dietary ME levels, respectively.

## 4. Discussion

Dietary treatments and nutrient specifications were designed carefully in order to be able to test our hypothesis. As outlined in the objectives of the experimental design it was intended that, all the experimental diets to be formulated with an optimal crude protein level while, suboptimal levels (restricted and low) of ME being isonitrogenous but not isocaloric, respectively as shown in Table 1. Our assumptions were based on the available literature [18] which presumed that dietary betaine would be capable of sparing an energy matrix value of 200 KJ/kg diet by conserving the energy required for oxidation of choline into betaine. Although the dietary ME levels were suboptimal (restricted and low ME) in either without or with betaine supplementation, respectively, production performance of Japanese quail has not been negatively impacted as shown in Table 2. Similar study [18] reported that dietary betaine with suboptimal levels of dietary ME did not induce a significant changes on growth performance of broilers chickens. However, other studies [4,34] demonstrated that, dietary betaine had a positive influence on poultry performance. It is worth mentioning that, dietary betaine effect is potentially influenced by the presence of other methyl donors in poultry diet and/or presence of stressors [35]. Therefore, the positive effect of dietary betaine on performance appeared clearly in experiment used methionine-deficient diet [13] and in stress based experiments [34]. 

Serum lipids levels were selected to be measured as an important indicator of lipid and energy metabolism in Japanese quail. The current study demonstrated the role of dietary betaine in increasing serum TAG and TC levels of quails fed diets containing restricted and low ME levels compared with that of birds fed betaine-non-supplemented diets containing optimum and restricted ME levels (Table 3). These results were consistent with previous work [18] stated the same role of dietary betaine in broilers at higher (2.0 g/kg), and lower (1.3 g/kg) levels of supplementation. The decreased levels of serum TAG might be attributed to the activity of hormone-sensitive lipase in response to dietary betaine. This hormone accelerates hydrolysis of TAG into free fatty acids and glycerol [10,35]. The current findings are consistent with previous report showed that dietary betaine reduced the serum lipids of broilers chickens subjected to chronic heat stress [35]. On the other hand, the reduction of both serum TAG and TC levels (Table 3) was attributed mainly to lowering ME levels especially in low energy diets either without or with betaine supplementation. The current findings are consistent with earlier study indicated that lower dietary ME levels reduced the fat deposition in White Pekin ducklings [36] due to reduction of activities of enzymes essential for catalysis of hepatic lipogenesis [37]. Moreover, earlier reports indicated that both quantitative and qualitative feed restriction induced an inhibition to hepatic lipogenesis and elevated the rate of *β*-oxidation [38]. The linear decrease in the values of serum HDL-c (*p* < 0.0001) shown in Table 3 was mainly attributed to the instant decreased level of ME intake. Similar results demonstrated that dietary betaine reduced serum TC and HDL-c in broiler chickens [10,35]. In the current study, the increased level of serum LDL-c and VLDL-c in birds fed betaine-supplemented diet containing optimum ME level compared to that of birds fed betaine non supplemented diet containing the same ME levels (Table 3) may attributed to the choline-sparing activity of dietary betaine. Choline-sparing activity of dietary betaine increased choline availability and accelerates fat removal from liver and prevents fatty liver [39]. Meanwhile, betaine as methyl donor increased the biosynthesis of carnitine via transmethylation reactions that enhances obligatory oxidation of long-chain fatty acids by facilitating their transport across the inner mitochondrial membrane [11]. 

Dietary betaine or different ME levels had no significant effects on breast meat ATP and AMP levels (Table 4). This might be explained by the compensatory mechanisms exerted by dietary betaine during nutritional imbalances especially in restricted and low energy diets, respectively. The significant increase of ADP in breast meat (*p* = 0.042) is a form of transient energy reserve ready to be utilized during post-mortem glycolysis in breast meat [15]. Dietary betaine showed successful antioxidant property. This has been observed in the form of lowered MDA level in breast meat (*p =* 0.002) of quails fed diet containing the optimum level of ME compared to that of birds fed diets containing restricted or low ME levels with dietary betaine and that of birds fed diets containing optimum and restricted ME levels without betaine supplementation (Table 4). Thus, the lowered MDA concentration was mainly attributed to the ameliorative antioxidant effect of dietary betaine against both the absence of betaine in restricted ME level diet and betaine supplemented with low ME level. The current results are consistent with the previous report [2] indicated that dietary betaine improved meat quality of broilers by reducing lipid peroxidation [15]. Recently, dietary betaine improved the antioxidant status of muscle of broilers under stress [40]. Antioxidants alleviated the oxidative stress in meat-type chickens by decreasing both lipid peroxidation and stabilizing oxidation in meat after slaughter [3]. The current study reported a significant reduction in TVBN of breast meat (*p* < 0.0001) of birds fed betaine-supplemented diets containing optimum ME levels compared to that of birds fed betaine-non-supplemented diet containing both optimum and low ME levels, respectively (Table 4). This might be attribute to the osmoprotectant property of dietary betaine on the muscular cells, which improved the keeping quality of meat. Earlier study indicated that dietary betaine protected the intestinal cells and cell membranes from the osmotic stress in broiler chickens [41]. The production of TVBN was mostly attributed to microbial and enzymatic decomposition of meat occurring during storage [42], and always used as an indicator for meat freshness [43]. The increased levels of TVBN in diets contain low ME levels irrespective to betaine supplementation (Table 4) was mainly attributed to the lower ME level which disable dietary betaine to protect the muscular cells. In addition, the presence of dietary betaine may promoted the microbial growth in breast meat which may exaggerated the increase in TVBN level in diet of low ME level (Table 4) [44]. 

The significant reduction of total lipids in breast meat in response to dietary betaine and/or lower ME levels may attributed to the well-known role of dietary betaine in lipid metabolism especially when added to diets containing low ME levels. This role of dietary betaine in lipid metabolism is mainly in the form of enhanced activity of both lipase and hormone-sensitive lipase [5]. Higher carcass yield and lower carcass fat are the principal objective of poultry industry as outlined recently [17]. Lower levels of total lipids of breast meat was highly noticeable in low ME level diets and betaine-non-supplemented diet (Table 5). These results are consistent with that observed earlier in poultry [17]. Although the nutritional status, dietary energy level in poultry and their metabolic consequences are the main factors regulating lipogenesis, the fat-reducing effect of nutritional factors has not been fully understood and need further investigation [17]. At optimum dietary ME levels, there was a significant reduction in breast-meat cholesterol of betaine-supplemented birds compared to the betaine-non-supplemented birds shown in Table 5. This may attributed to enhanced betaine activity in the reduction of serum cholesterol (Table 3) and consequent breast-meat cholesterol (Table 5). The current results are consistent with the earlier reports which revealed that dietary betaine decreased serum LDL-c and HDL-c in broiler’s carcass subjected to chronic heat stress [35] and decreased serum level of cholesterol in laying hens [45]. In the current study, ME restriction in betaine-non- supplemented diets also was highly effective to reduce the cholesterol content of breast meat as a major consequences of lower meat total lipid. The current results were consistent with previous study in which both qualitative and quantitative feed restriction had significantly reduced body fat content in broilers, meat-type ducks, and broiler breeders [46]. The reduced fat deposited in poultry due to feed restriction possibly accomplished by inhibiting hepatic lipogenesis and enhanced *β*-oxidation of fatty acids. Thus, restricted feed consumption in the intensive poultry production system could be a successful tool for reducing the level of undesirable fat in modern strains of poultry [17]. The current data revealed that, the dietary betaine significantly increased the concentrations of both EPA and DHA (*p* < 0.0001 and *p* = 0.0002), respectively in breast meat of birds fed betaine-supplemented diets compared to that of birds fed betaine-non-supplemented diets (Table 5). The current data are consistent with previous report [47] observed higher concentrations of PUFA in meat of growing lambs as a result of dietary supplementation of rumen-protected betaine. In the current study, the significant decreased level of lysine in breast meat of birds fed betaine-supplemented diets compared to that of birds fed betaine-non-supplemented diets shown in Table 6 was mainly attributed to the enhanced utilization of free essential amino acids in protein biosynthesis in response to dietary betaine. Whilst, the exploitation of dietary sulfur-containing amino acids for protein biosynthesis does not affect the level of methionine and cysteine which might be attributed to the methionine-sparing activity of dietary betaine. This achieved by the higher methionine and cysteine availability for higher protein biosynthesis [13]. Meanwhile, the accelerated utilization of amino acids for such higher protein biosynthesis on the expense of fats in muscular tissue has resulted in fewer amino acids for deamination and consequent lower fat biosynthesis [48]. This induced lower levels of lysine in meat of birds fed betaine-supplemented diets in the current study. In addition, lower levels of lysine in the current study possibly because lysine contributed with methionine and betaine in the synthesis of _L_-carnitine that act as facilitator of fatty acids oxidation in the liver [49]. For the free nonessential amino acids, the significant increase in glutamine level in meat of birds fed betaine-supplemented diet compared to that of birds fed betaine-non-supplemented diets (Table 6) may attributed to the less available lysine which contributes in protein as well as _L_-carnitine biosynthesis. the current results are consistent with earlier report which proved that free glutamate content in chickens’ meat increased due to the reduction of dietary lysine [50]. The higher level of free glutamic acid in chickens’ meat had a great impact on the improvement of taste [51].

Similar pH_U_ values in breast meat (Table 7) revealed that neither glycolysis nor lactic acid accumulation in breast meat has been affected by either betaine or reduced ME levels [40]. Noteworthy, the low ME level in diet supplemented with betaine has improved the WHC of breast meat (Table 7). The pronounced improvement of breast meat WHC (i.e., higher tenderness) was accompanied with desirable reduction of both cooking and thawing losses (Table 7) of low ME level in diet supplemented with betaine. This means that, betaine role was effective especially with the low energy level to improve the WHC which could improves the tenderness of breast meat possibly by significant reduction of both cooking and thawing losses. The current results were consistent with previous work showed that, betaine supplementation can improve the quality of meat by reducing carcass drip loss [52], although others indicated that dietary betaine had no effect on the quality of meat [53]. Our findings proposed that betaine could lessen the negative impacts of the decreased ME on Japanese quail meat which was similar to the benefits of betaine supplementation in broiler chickens under heat stress [40]. The minimum moisture percentage of breast meat was observed in meat of quails fed betaine-supplemented diets containing low dietary ME level (Table 7). Hence increasing total dry matter especially protein content of quail’s meat might compensate weight gain in in quails fed diets containing low ME levels. In contrast, recent work [40] found that, betaine supplementation did not affect moisture content of broilers’ meat. These discrepancies in results might indicate that, possible long scale future studies may be accomplished. Average Moisture content of Japanese quail meat reported to be around 72.35 ± 0.4 at six weeks of age and 69.87 ± 0 at 8 month of age [54]. Dietary betaine did not alters ash content among all dietary treatments. This was in accordance with those previously reported results in broilers [2].

## 5. Conclusions

From the results of this study we could conclude that, neither betaine nor energy levels has impaired performance, energy metabolism of muscle due to energy-sparing activity of betaine which had significantly compensated the restricted and low energy levels. Dietary betaine induced significant reduction in serum TAG and TC of Japanese quail fed diets containing either restricted or low ME. Dietary betaine induced significant increase in serum LDL-c and VLDL-c of Japanese quail fed diet containing optimum ME levels. Dietary betaine induced significant reduction in serum LDL-c and VLDL-c of Japanese quail fed diet containing restricted or low ME levels. Muscular cellular energy was maintained by betaine supplementation even with restricted and low energy levels. Dietary betaine could improve the antioxidant stability (i.e., lower MDA level) and meat quality characteristics (i.e., lower total lipids while, higher omega-3 PUFA and tenderness) of breast meat from quails fed diets containing restricted and low ME. Moreover, cooking loss (CL), thawing loss (ThL) and water holding capacity (WHC) were improved in the breast meat of quails fed betaine supplemented diets containing low ME. Dietary betaine had significantly reduced breast meat total lipids of birds fed diets containing low ME level compared with that of birds fed either betaine-supplemented or betaine non-supplemented diets containing optimum and restricted ME levels. Breast meat cholesterol was reduced in birds fed betaine-supplemented diets with an optimum, restricted and low ME levels compared with that of birds fed betaine-non-supplemented diet with optimum ME level. Dietary betaine improves meat quality of Japanese quail fed either diets containing either restricted or low ME by enrichments the meat with omega-3 fatty acids and reduction of lipids levels. Further investigation of another dose for betaine in low energy diets is needed to fully understand the lipid metabolism during such nutritional imbalances.

## Figures and Tables

**Table 1:** Ingredients composition, calculated analysis (g/100 g as fed basis unless otherwise indicated) of experimental diets.

Ingredients (%)	^1^ Betaine Levels					
	0%	0.15%	0%	0.15%	0%	0.15%
Corn grain (yellow, crushed)	54.50	54.50	50.19	50.19	46.12	46.12
^2^ Soybean meal solvent extracted 44%	35.70	35.70	34.00	34.00	34.00	34.00
^3^ Corn gluten meal 60%	6.00	6.00	6.30	6.30	5.70	5.70
Wheat bran	0.78	0.78	6.50	6.50	11.23	11.23
Dicalcium phosphate	1.11	1.11	1.10	1.10	1.02	1.02
Lime stone	1.10	1.10	1.08	1.08	1.10	1.10
Sodium chloride	0.30	0.30	0.30	0.30	0.30	0.30
^4^ Vitamins and Mineral Mix	0.30	0.30	0.30	0.30	0.30	0.30
_L_-Lysine HCl	0.10	0.10	0.13	0.13	0.11	0.11
DL-Methionine	0.11	0.11	0.10	0.10	0.11	0.11
Total (%)	100.00	100.00	100.00	100.00	100.00	100.00
Calculated analysis						
^5^ ME (kcal/kg diet)	2900	2900	2800	2800	2700	2700
Crude protein (CP)%	24	24	24	24	24	24
Methionine%	0.5	0.5	0.5	0.5	0.5	0.5
lysine	1.3	1.3	1.3	1.3	1.3	1.3
Ca%	0.8	0.8	0.8	0.8	0.8	0.8
Avail *P*%	0.3	0.3	0.3	0.3	0.3	0.3

^1^ Betaine supplemented to the diets over the top (1.5 g/kg). ^2^ Soybean meal solvent extract contains 44% crude protein. ^3^ Corn gluten meal contains 60% crude protein. ^4^ Vitamin and mineral premix supplied each kg of feeds with: Vitamin A 12,000 IU; vitamin D_3_ 2000 IU; vitamin E 10 mg; vitamin K_3_ 2 mg; vitamin B_1_ 1 mg; vitamin B_2_ 5 mg; vitamin B_6_ 1.5 mg; vitamin B_12_ 0.01 mg; Biotin 0.05 mg; pantothenic acid 10 mg; Nicotinic acid 30 mg; Folic acid 1 mg; Manganese 60 mg; Iron 30 mg; Copper 10 mg; Iodine 1 mg; Selenium 0.01 mg; Cobalt 0.01 mg. ^5^ ME: metabolizable energy. The crude protein for every treatment was analyzed by Vapodest 30 S distillation unit, Gerhardet, Germany. The results for crude protein analysis were nearly the same for each treatment and was around 23.81%. This gives us an indication that there were no mixing errors.

**Table 2:** Effect of dietary betaine supplementation and energy levels on performance of Japanese quail.

Parameters	0% Betaine			0.15% Betaine			SEM	*p* Values		
	Optimum	Restricted	Low	Optimum	Restricted	Low		Betaine	ME	Betaine × ME
Initial weight (g)	45.5	45.41	45.58	45.58	45.5	45.5	0.072	0.851	0.864	0.864
Final weight (g)	126.6	127.34	134.66	128.42	123.37	123.53	1.983	0.286	0.747	0.435
WG (g/35 day)	81.1	81.92	89.08	82.84	77.86	78.02	1.997	0.286	0.76	0.448
FI (g/35 day)	363.04	352.06	408.4	354.21	365.88	352.93	6.357	0.21	0.304	0.117
FCR (g/g)	4.47	4.34	4.58	4.28	4.69	4.57	0.08	0.733	0.587	0.392

ME; metabolizable energy; SEM: standard error of means; WG: weight gain; FI: feed intake; FCR: feed conversion ratio; Optimum: 2900 kcal ME/kg; Restricted: 2800 kcal ME/kg; Low: 2700 kcal ME/kg.

**Table 3:** Effect of dietary betaine supplementation and energy levels on serum lipids of Japanese quail.

Parameters	0% Betaine			0.15% Betaine			SEM	*p* Value		
	Optimum	Restricted	Low	Optimum	Restricted	Low		Betaine	ME	Betaine × ME
Triacylglycerol (mg/dL)	131.83 ^b^	133.75 ^b^	115.41 ^c^	141.75 ^a^	103.75 ^c^	103.85 ^c^	1.482	0.0013	<0.0001	<0.0001
Total cholesterol (mg/dL)	150.65 ^a^	161.58 ^a^	128.00 ^b^	164.00 ^a^	117.35 ^b^	121.90 ^b^	2.058	0.005	<0.0001	<0.0001
HDL-cholesterol (mg/dL)	50.15 ^a^	48.05 ^a^	37.46 ^c^	49.83 ^a^	38.50 ^c^	37.31 ^c^	1.003	0.106	<0.0001	0.108
LDL-cholesterol (mg/dL)	74.11 ^b^	86.75 ^a^	67.50 ^b^	85.81 ^a^	58.06 ^b^	63.81 ^b^	1.794	0.064	0.01	0.0003
VLDL-cholesterol (mg/dL)	14.82 ^b^	17.35 ^a^	13.49 ^b^	17.16 ^a^	11.62 ^b^	12.76 ^b^	0.359	0.064	0.01	0.0003
Total cholesterol/HDL	3.04	3.4	3.45	3.3	3.1	3.29	0.067	0.613	0.476	0.213
LDL/HDL	1.5	1.84	1.83	1.72	1.54	1.73	0.058	0.628	0.519	0.198

^a–c^ Means with different letters superscripts at the same row differ significantly at (*p* ≤ 0.05). SEM: standard error of means; ME; metabolizable energy; HDL: high density lipoprotein-cholesterol; LDL: low density lipoprotein-cholesterol; VLDL: very low-density lipoprotein-cholesterol. Optimum: 2900 kcal ME/kg; Restricted: 2800 kcal ME/kg; Low: 2700 kcal ME/kg.

**Table 4:** Effect of dietary betaine supplementation and energy levels on cell energy, lipid peroxidation and protein breakdown of breast meat of Japanese quail.

Parameters	0% Betaine			0.15% Betaine			SEM	*p* Value		
	Optimum	Restricted	Low	Optimum	Restricted	Low		Betaine	ME	Betaine × ME
ATP (μg/g meat)	14.93	15.63	12.54	17.03	15.44	13.33	0.589	0.461	0.115	0.735
ADP (μg/g meat)	7.07	7.97	7.46	8.06	9.77	8.88	0.309	0.042	0.265	0.871
AMP (μg/g meat)	6.21	5.65	4.72	5.57	5.92	5.04	0.229	0.968	0.183	0.638
MDA (nmol/g meat)	12.04 ^ab^	14.14 ^a^	8.98 ^b^	10.65 ^b^	12.24 ^ab^	14.57 ^a^	0.358	0.304	0.131	0.002
TVBN (mg/100 g meat)	27.59 ^a^	18.95 ^b^	24.11 ^a^	19.00 ^c^	23.77 ^b^	26.71 ^a^	0.439	0.668	0.009	<0.0001

^a–c^ Means with different letters superscripts at the same row differ significantly at (*p* ≤ 0.05). SEM: standard error of means; ME: metabolizable energy; ATP: adenosine triphosphate; ADP: adenosine diphosphate; AMP; adenosine monophosphate: MDA: malondialdehyde; TVBN: total volatile basic nitrogen; Optimum: 2900 kcal ME/kg; Restricted: 2800 kcal ME/kg; Low: 2700 kcal ME/kg.

**Table 5:** Effect of dietary betaine supplementation and energy levels on breast meat fat, cholesterols and fatty acids compositions of Japanese quail.

Parameters	0% Betaine			0.15% Betaine			SEM	*p* Value		
	Optimum	Restricted	Low	Optimum	Restricted	Low		Betaine	ME	Betaine × ME
Total lipids (g/100 g meat)	8.56 ^a^	9.10 ^a^	7.08 ^b^	8.75 ^a^	9.12 ^a^	7.06 ^b^	0.113	0.009	0.006	0.009
Cholesterol (mg/100 g meat)	81.12 ^a^	70.52 ^ab^	59.36 ^c^	75.45 ^a^	77.03 ^a^	78 ^a^	1.184	0.017	0.012	0.006
Palmitoleic Acid (C16:1)	6.4	6.31	6.75	6.61	6.6	6.79	0.113	0.436	0.502	0.893
Linoleic Acid (C18:2)	13.54	14.54	14.95	14.9	14.01	13.26	0.291	0.629	0.971	0.138
Linolenic Acid (C18:3)	0.34	0.33	0.29	0.31	0.32	0.33	0.01	0.927	0.844	0.442
Arachidonic Acid (C20:4)	3.99	4.17	3.88	3.81	4.18	4.41	0.057	0.325	0.142	0.068
EPA (C20:5)	0.16	0.16	0.17	0.27	0.28	0.24	0.006	<0.0001	0.547	0.261
DHA (C22:6)	0.91	1.03	1.14	1.63	1.81	1.46	0.058	0.0002	0.553	0.255

^a–c^ Means with different letters superscripts at the same row differ significantly at (*p* ≤ 0.05). SEM: standard error of means; ME: metabolizable energy; EPA; Ecosapenaenoic Acid; DHA: Docosahexaenoic Acid; Optimum: 2900 kcal ME/kg; Restricted: 2800 kcal ME/kg; Low: 2700 kcal ME/kg.

**Table 6:** Effect of dietary betaine supplementation and energy levels on breast meat amino acid composition of (nmol/g meat) Japanese quail.

Parameters	0% Betaine			0.15% Betaine			SEM	*p* Value		
	Optimum	Restricted	Low	Optimum	Restricted	Low		Betaine	ME	Betaine × ME
Essential amino acids										
Thrionine	1.16	1.35	1.2	1.35	1.48	1.23	0.043	0.192	0.17	0.777
Histidine	1.36	1.29	1.2	1.22	1.31	1.31	0.033	0.953	0.819	0.326
Valine	1.23	1.23	1.25	1.31	1.31	1.22	0.03	0.482	0.886	0.685
Methionine	1.22	1.16	1.12	1.33	1.26	1.24	0.034	0.129	0.501	0.99
Arginine	1.15	1.14	1.2	1.18	1.31	1.37	0.034	0.099	0.417	0.643
Leucine	1.19	1.25	1.19	1.14	1.19	1.19	0.036	0.596	0.846	0.928
Isoleucine	1.18	1.27	1.3	1.23	1.33	1.13	0.039	0.791	0.576	0.415
Phenylalanine	1.16	1.32	1.08	1.19	1.33	1.22	0.035	0.433	0.13	0.706
Lysine	1.19	1.2	1.05	0.93	0.84	0.72	0.057	0.017	0.439	0.941
Nonessential amino acids										
Asparagine	2.56	2.89	2.68	2.94	2.76	2.82	0.076	0.424	0.903	0.411
Glycine	1.18	1.44	1.3	1.2	1.3	1.07	0.043	0.193	0.186	0.47
Glutamine	1.22	1.3	1.27	2.41	2.03	2.23	0.061	<0.0001	0.619	0.353
Serine	1.17	1.18	1.19	1.18	1.25	1.18	0.032	0.667	0.866	0.874
Proline	1.25	1.45	1.29	1.33	1.27	1.42	0.035	0.892	0.657	0.197

SEM: standard error of means; ME; metabolizable energy; Optimum: 2900 kcal ME/kg; Restricted: 2800 kcal ME/kg; Low: 2700 kcal ME/kg.

**Table 7:** Effect of dietary betaine supplementation and energy levels on physicochemical characters of breast meat of Japanese quail.

Parameters	0% Betaine			0.15% Betaine			SEM	*p* Value		
	Optimum	Restricted	Low	Optimum	Restricted	Low		Betaine	ME	Betaine × ME
pH_U_	5.82	5.85	5.85	5.86	5.86	5.92	0.015	0.161	0.521	0.694
WHC%	76.3	71.11	77.21	72.82 ^b^	70.43 ^b^	85.54 ^a^	0.729	0.483	<0.0001	0.027
CL%	15.35	14.26	18.69	17.06 ^ab^	19.23 ^a^	10.76 ^b^	0.834	0.806	0.606	0.021
ThL%	5.03 ^b^	6.21 ^b^	12.15 ^a^	8.11	8.21	5.49	0.526	0.625	0.239	0.005
Moisture%	78.24 ^ab^	80.36 ^a^	74.93 ^b^	78.43 ^a^	80.95 ^a^	69.22 ^b^	0.857	0.356	0.004	0.28
Ash%	1.4	1.58	1.31	1.51	1.68	1.4	0.058	0.394	0.184	0.996

^a,b^ Means with different letters superscripts at the same row differ significantly at (*p* ≤ 0.05). SEM: standard error of means; ME: metabolizable energy; pHu: ultimate PH; WHC: water holding capacity; CL: cooking loss; ThL: thawing loss; Optimum: 2900 kcal ME/kg; Restricted: 2800 kcal ME/kg; Low: 2700 kcal ME/kg.

## Data Availability

The data presented in this study are available on request from the corresponding author.
